# Supplementary material for: The Life Cycle of Aurelia aurita Depends on the Presence of a Microbiome in Polyps Prior to Onset of Strobilation
Source: Microbiol Spectr. 2023 Jun 28;11(4):e00262-23. doi: 10.1128/spectrum.00262-23 (PMC10433978; doi:10.1128/spectrum.00262-23)
Supplement: Supplemental file 1 — Supplemental material. Download spectrum.00262-23-s0001.pdf, PDF file, 0.8 MB [file spectrum.00262-23-s0001.pdf]

Title

Asexual reproduction of *Aurelia aurita* depends on the presence of a balanced microbiome at polyp stage

Jensen N.[1], Weiland-Bräuer N.[1], Joel S.[1], Chibani C.M.[1], Schmitz R.A.[1]*

[1] Institute of General Microbiology, Christian-Albrechts University Kiel, Am Botanischen Garten 1-9, 24118, Kiel, Germany

* rschmitz@ifam.uni-kiel.de

Keywords: *Aurelia aurita*, native microbiota, reproduction, recolonization, life cycle

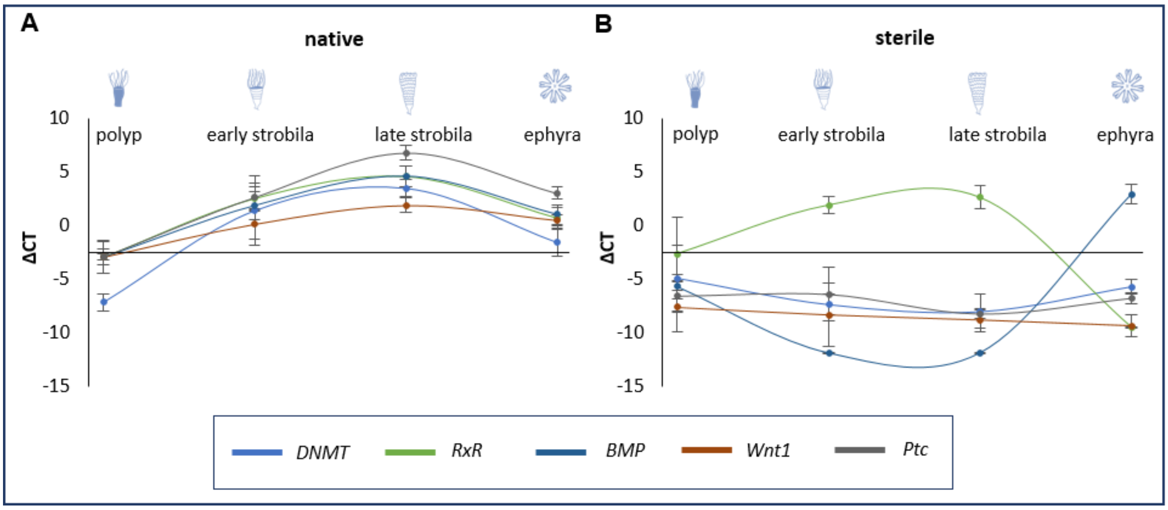

Fig. S 1: Impact of the native microbiota on the transcription of generally conserved developmental genes during asexual reproduction. Gene expression (ΔCt values) was monitored by qRT-PCR in polyps, early strobilae, late strobilae, and ephyrae in native (A) and sterile (B) animals.

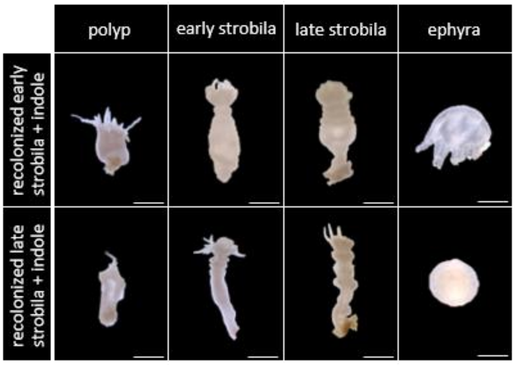

Fig. S 2: Recolonization in constant presence of inducer does not restore defective phenotypes. When recolonization of sterile early or late strobilae was performed with a constant supply of inducer, this did not prevent the formation of malformed strobilae, inadequate segmentation and strongly deformed ephyra**.**

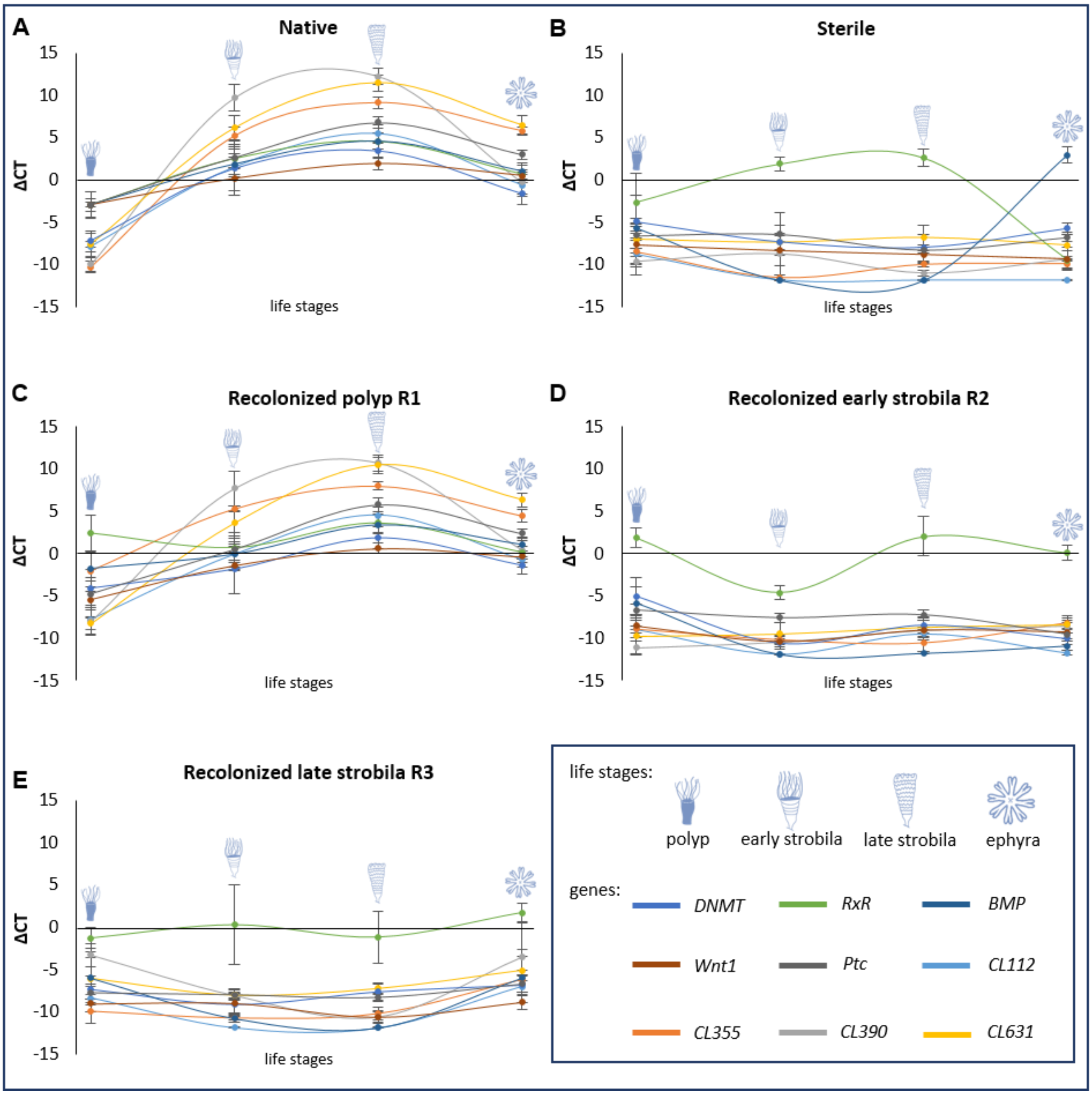

Fig. S 3: Gene expression profiles for all assessed treatments during development over time. The expression (ΔCt values) of the four *A. aurita*-specific strobilation genes and the five generally conserved developmental genes are shown for **A**: native animals, **B**: sterile animals, **C**: recolonized polyps, **D**: recolonized early strobilae), and **E**: recolonized late strobilae.

Tab. S 1: Average (Δ) CT values and the respective standard abbreviation of 6 replicates.

Treatment	Time Point	Gene	Average CT	Average ΔCT	Standard Abbreviation
Native	T0	CL112	36,007	-7,88	0,63
Native	T4	CL112	26,607	1,50	1,16
Native	T9	CL112	22,661	5,44	1,00
Native	T12	CL112	28,726	-0,62	1,29
Native	T0	CL355	38,454	-10,34	0,68
Native	T4	CL355	22,903	5,20	0,97
Native	T9	CL355	18,97	9,13	0,66
Native	T12	CL355	16,296	5,81	0,47
Native	T0	CL390	38,071	-9,97	0,91
Native	T4	CL390	18,437	9,67	1,57
Native	T9	CL390	15,906	12,20	0,99
Native	T12	CL390	28,396	-0,29	1,43
Native	T0	CL631	35,69	-7,59	1,56
Native	T4	CL631	21,994	6,11	1,43
Native	T9	CL631	16,659	11,44	0,95
Native	T12	CL631	18,647	6,46	1,09
Native	T0	DNMT	35,263	-7,16	0,82
Native	T4	DNMT	26,708	1,40	3,24
Native	T9	DNMT	24,63	3,47	0,83
Native	T12	DNMT	29,699	-1,60	1,25
Native	T0	RXR	31,047	-2,94	1,55
Native	T4	RXR	25,57	2,53	1,11
Native	T9	RXR	23,526	4,58	0,92
Native	T12	RXR	27,407	0,70	0,98
Native	T0	BMP	32,259	-2,94	1,46
Native	T4	BMP	26,247	1,86	1,27
Native	T9	BMP	23,316	4,58	0,92
Native	T12	BMP	27,081	1,02	0,91
Native	T0	Wnt1	33,45	-2,94	0,76
Native	T4	Wnt1	27,977	0,13	1,38
Native	T9	Wnt1	26,231	1,87	0,69
Native	T12	Wnt1	27,623	0,48	0,48
Native	T0	Ptc	32,906	-2,94	0,25
Native	T4	Ptc	25,467	2,64	1,33
Native	T9	Ptc	21,328	6,78	0,71
Native	T12	Ptc	25,119	2,98	0,57
Sterile	T0	CL112	36,473	-8,77	2,40
Sterile	T4	CL112	39,975	-11,87	0,04
Sterile	T9	CL112	39,98	-11,88	0,04
Sterile	T12	CL112	39,982	-11,88	0,04
Sterile	T0	CL355	38,013	-8,49	1,32
Sterile	T4	CL355	39,643	-11,54	0,26

Treatment	Time Point	Gene	Average CT	Average ΔCT	Standard Abbreviation
Sterile	T9	CL355	38,076	-9,97	0,27
Sterile	T12	CL355	37,981	-9,88	0,62
Sterile	T0	CL390	31,414	-9,63	0,64
Sterile	T4	CL390	36,812	-8,71	1,42
Sterile	T9	CL390	39,121	-11,02	0,38
Sterile	T12	CL390	37,464	-9,36	1,36
Sterile	T0	CL631	34,171	-7,03	1,56
Sterile	T4	CL631	35,480	-7,38	0,80
Sterile	T9	CL631	34,921	-6,82	1,49
Sterile	T12	CL631	35,823	-7,72	1,30
Sterile	T0	DNMT	33,035	-4,93	3,11
Sterile	T4	DNMT	35,47	-7,37	1,02
Sterile	T9	DNMT	36,126	-8,02	1,61
Sterile	T12	DNMT	33,832	-5,73	0,70
Sterile	T0	RXR	30,758	-2,65	3,41
Sterile	T4	RXR	26,208	1,90	0,80
Sterile	T9	RXR	25,488	2,62	1,06
Sterile	T12	RXR	37,616	-9,51	0,08
Sterile	T0	BMP	33,811	-5,71	1,14
Sterile	T4	BMP	39,95	-11,90	0,07
Sterile	T9	BMP	39,962	-11,90	0,07
Sterile	T12	BMP	25,177	2,93	0,93
Sterile	T0	Wnt1	35,725	-7,62	2,33
Sterile	T4	Wnt1	36,445	-8,34	2,93
Sterile	T9	Wnt1	36,928	-8,82	1,08
Sterile	T12	Wnt1	37,48	-9,38	1,02
Sterile	T0	Ptc	34,687	-6,58	1,45
Sterile	T4	Ptc	34,518	-6,41	2,50
Sterile	T9	Ptc	36,386	-8,28	0,38
Sterile	T12	Ptc	34,889	-6,79	0,54
Recolonized Polyp	T0	CL112	36,169	-7,79	1,67
Recolonized Polyp	T4	CL112	28,217	-0,11	1,52
Recolonized Polyp	T9	CL112	23,583	-4,52	0,99
Recolonized Polyp	T12	CL112	28,842	-0,74	0,81
Recolonized Polyp	T0	CL355	30,159	-2,06	2,41
Recolonized Polyp	T4	CL355	22,82	5,28	0,39
Recolonized Polyp	T9	CL355	20,125	7,98	0,47
Recolonized Polyp	T12	CL355	23,651	4,45	0,8
Recolonized Polyp	T0	CL390	36,126	-8,02	1,58
Recolonized Polyp	T4	CL390	20,419	7,68	2,02
Recolonized Polyp	T9	CL390	17,406	10,70	0,97
Recolonized Polyp	T12	CL390	27,674	0,43	1,11
Recolonized Polyp	T0	CL631	25,406	-8,27	0,77
Recolonized Polyp	T4	CL631	24,527	3,58	1,48
Recolonized Polyp	T9	CL631	17,671	10,43	0,94

Treatment	Time Point	Gene	Average CT	Average ΔCT	Standard Abbreviation
Recolonized Polyp	T12	CL631	21,752	6,35	0,83
Recolonized Polyp	T0	DNMT	32,223	-4,12	2,24
Recolonized Polyp	T4	DNMT	29,951	-1,85	2,94
Recolonized Polyp	T9	DNMT	26,202	1,90	1,28
Recolonized Polyp	T12	DNMT	29,494	-1,39	0,98
Recolonized Polyp	T0	RXR	25,722	2,38	2,17
Recolonized Polyp	T4	RXR	27,44	0,66	1,76
Recolonized Polyp	T9	RXR	24,508	3,60	1,11
Recolonized Polyp	T12	RXR	27,997	0,11	0,78
Recolonized Polyp	T0	BMP	29,874	-1,77	2,12
Recolonized Polyp	T4	BMP	28,169	-0,07	1,88
Recolonized Polyp	T9	BMP	24,688	3,42	1,07
Recolonized Polyp	T12	BMP	27,013	1,09	0,80
Recolonized Polyp	T0	Wnt1	33,547	-5,44	2,22
Recolonized Polyp	T4	Wnt1	29,516	-1,41	0,67
Recolonized Polyp	T9	Wnt1	27,505	0,60	0,58
Recolonized Polyp	T12	Wnt1	28,49	-0,39	0,71
Recolonized Polyp	T0	Ptc	32,891	-4,79	1,92
Recolonized Polyp	T4	Ptc	27,628	0,48	0,36
Recolonized Polyp	T9	Ptc	22,352	5,75	0,80
Recolonized Polyp	T12	Ptc	25,76	2,34	0,55
Recolonized early Strobila	T0	CL112	36,875	-8,93	0,06
Recolonized early Strobila	T4	CL112	39,984	-11,88	0,04
Recolonized early Strobila	T9	CL112	37,56	-9,46	0,51
Recolonized early Strobila	T12	CL112	39,859	-11,76	0,26
Recolonized early Strobila	T0	CL355	36,593	-8,98	0,35
Recolonized early Strobila	T4	CL355	38,306	-10,20	0,39
Recolonized early Strobila	T9	CL355	38,664	-10,56	1,01
Recolonized early Strobila	T12	CL355	36,331	-8,23	0,6
Recolonized early Strobila	T0	CL390	37,737	-11,11	0,81
Recolonized early Strobila	T4	CL390	38,566	-10,46	0,12
Recolonized early Strobila	T9	CL390	37,246	-9,14	0,41
Recolonized early Strobila	T12	CL390	36,739	-8,64	0,8
Recolonized early Strobila	T0	CL631	35,133	-9,74	2,1
Recolonized early Strobila	T4	CL631	37,422	-9,46	1,33
Recolonized early Strobila	T9	CL631	36,787	-8,68	1,1
Recolonized early Strobila	T12	CL631	36,48	-8,38	1,01
Recolonized early Strobila	T0	DNMT	33,155	-5,05	2,22
Recolonized early Strobila	T4	DNMT	38,671	-10,57	0,43
Recolonized early Strobila	T9	DNMT	36,559	-8,46	0,97
Recolonized early Strobila	T12	DNMT	38,152	-10,05	0,72
Recolonized early Strobila	T0	RXR	26,204	1,90	1,17
Recolonized early Strobila	T4	RXR	32,732	-4,63	0,84
Recolonized early Strobila	T9	RXR	26,081	2,02	2,30
Recolonized early Strobila	T12	RXR	28,05	0,05	0,87

Treatment	Time Point	Gene	Average CT	Average ΔCT	Standard Abbreviation
Recolonized early Strobila	T0	BMP	33,984	-5,88	1,97
Recolonized early Strobila	T4	BMP	39,981	-11,90	0,03
Recolonized early Strobila	T9	BMP	39,861	-11,76	0,14
Recolonized early Strobila	T12	BMP	39,668	-10,90	0,49
Recolonized early Strobila	T0	Wnt1	36,65	-8,55	1,37
Recolonized early Strobila	T4	Wnt1	38,564	-10,46	0,81
Recolonized early Strobila	T9	Wnt1	37,147	-9,04	1,20
Recolonized early Strobila	T12	Wnt1	37,372	-9,27	1,05
Recolonized early Strobila	T0	Ptc	34,784	-6,68	0,73
Recolonized early Strobila	T4	Ptc	35,701	-7,60	0,55
Recolonized early Strobila	T9	Ptc	35,355	-7,25	0,60
Recolonized early Strobila	T12	Ptc	37,661	-9,56	0,53
Recolonized late Strobila	T0	CL112	37,037	-8,37	1,58
Recolonized late Strobila	T4	CL112	39,972	-11,87	0,07
Recolonized late Strobila	T9	CL112	39,982	-11,88	0,04
Recolonized late Strobila	T12	CL112	35,143	-7,04	1,91
Recolonized late Strobila	T0	CL355	37,087	-9,91	1,45
Recolonized late Strobila	T4	CL355	38,812	-10,71	0,25
Recolonized late Strobila	T9	CL355	38,291	-10,19	0,77
Recolonized late Strobila	T12	CL355	34,301	-6,20	2,71
Recolonized late Strobila	T0	CL390	36,195	-3,31	1,32
Recolonized late Strobila	T4	CL390	36,195	-8,09	0,29
Recolonized late Strobila	T9	CL390	38,726	-10,62	0,6
Recolonized late Strobila	T12	CL390	31,665	-3,56	4,1
Recolonized late Strobila	T0	CL631	37,843	-6,07	3,17
Recolonized late Strobila	T4	CL631	36,114	-8,01	0,69
Recolonized late Strobila	T9	CL631	35,342	-7,24	0,65
Recolonized late Strobila	T12	CL631	33,239	-5,14	2,57
Recolonized late Strobila	T0	DNMT	35,445	-7,34	1,60
Recolonized late Strobila	T4	DNMT	37,229	-9,13	1,48
Recolonized late Strobila	T9	DNMT	35,735	-7,63	0,96
Recolonized late Strobila	T12	DNMT	34,947	-6,84	1,23
Recolonized late Strobila	T0	RXR	29,389	-1,29	1,23
Recolonized late Strobila	T4	RXR	27,829	0,27	4,72
Recolonized late Strobila	T9	RXR	29,287	-1,18	3,06
Recolonized late Strobila	T12	RXR	26,403	1,70	1,09
Recolonized late Strobila	T0	BMP	34,13	-6,03	2,41
Recolonized late Strobila	T4	BMP	38,888	-10,79	0,46
Recolonized late Strobila	T9	BMP	39,975	-11,90	0,04
Recolonized late Strobila	T12	BMP	34,113	-6,01	0,27
Recolonized late Strobila	T0	Wnt1	37,137	-9,03	1,03
Recolonized late Strobila	T4	Wnt1	37,087	-8,98	1,15
Recolonized late Strobila	T9	Wnt1	38,762	-10,66	0,72
Recolonized late Strobila	T12	Wnt1	36,955	-8,85	0,92
Recolonized late Strobila	T0	Ptc	35,841	-7,74	0,78

Treatment	Time Point	Gene	Average CT	Average ΔCT	Standard Abbreviation
Recolonized late Strobila	T4	Ptc	36,089	-7,99	0,57
Recolonized late Strobila	T9	Ptc	36,411	-8,31	0,43
Recolonized late Strobila	T12	Ptc	34,773	-6,67	1,05

Tab. S 2: Student's t-tests. Determination of statistical significance between the mean of Ct-values per gene per group.

life stage	treatment1	treatment2	n1	n2	p	p.adj	p.adj.signif
Segments	native	recolonized early strobila	89	86	4.74e-48	4.74e-47	****
Segments	native	recolonized late strobila	89	84	3.59e-49	3.59e-48	****
Segments	native	recolonized polyp	89	94	0.79	1	ns
Segments	native	sterile	89	89	8.13e-47	8.13e-46	****
Segments	recolonized early strobila	recolonized late strobila	86	84	0.397	1	ns
Segments	recolonized early strobila	recolonized polyp	86	94	3.65e-40	3.65e-39	****
Segments	recolonized early strobila	sterile	86	89	0.356	1	ns
Segments	recolonized late strobila	recolonized polyp	84	94	3.21e-41	3.21e-40	****
Segments	recolonized late strobila	sterile	84	89	0.082	0.825	ns
Segments	recolonized polyp	sterile	94	89	5.46e-39	5.46e-38	****
Ephyrae	native	recolonized early strobila	89	86	7.87e-60	7.87e-59	****
Ephyrae	native	recolonized late strobila	89	84	4.7e-61	4.7e-60	****
Ephyrae	native	recolonized polyp	89	94	0.362	1	ns
Ephyrae	native	sterile	89	89	2.63e-60	2.63e-59	****
Ephyrae	recolonized early strobila	recolonized late strobila	86	84	0.245	1	ns
Ephyrae	recolonized early strobila	recolonized polyp	86	94	1.14e-58	1.14e-57	****
Ephyrae	recolonized early strobila	sterile	86	89	0.024	0.243	ns
Ephyrae	recolonized late strobila	recolonized polyp	84	94	7.45e-60	7.45e-59	****
Ephyrae	recolonized late strobila	sterile	84	89	0.316	1	ns
Ephyrae	recolonized polyp	sterile	94	89	3.2e-59	3.2e-58	****
